# Totally single-incision laparoscopic choledochal cyst excision with intracorporeal hand-sewn Roux-en-Y reconstruction in children: clinical outcomes and learning curve

**DOI:** 10.3389/fped.2026.1951655

**Published:** 2026-10-14

**Authors:** Miao Luo, Zhijie Qin, Yingming Tang, Yize Zhuang

**Affiliations:** Department of Pediatric General Surgery, Shanghai Children’s Medical Center Guizhou Hospital, Shanghai Jiao Tong University School of Medicine, Guiyang, China

**Keywords:** children, choledochal cyst, CUSUM analysis, Roux-en-Y reconstruction, single-incision laparoscopy

## Abstract

**Objective:**

To evaluate the feasibility, perioperative outcomes, and learning curve of totally single-incision laparoscopic choledochal cyst excision with intracorporeal hand-sewn Roux-en-Y reconstruction in children.

**Methods:**

We retrospectively reviewed children who underwent laparoscopic choledochal cyst excision between January 2018 and December 2024. Thirty-five children underwent totally single-incision laparoscopic intracorporeal hand-sewn reconstruction (TSILC), and 40 underwent conventional single-incision laparoscopic-assisted reconstruction (SILC). Baseline characteristics, operative outcomes, recovery, complications, and follow-up were compared. Multivariable linear regression with HC3 robust standard errors was used to adjust continuous outcomes for baseline differences. The TSILC learning curve was assessed using cumulative sum (CUSUM) analysis of jejunojejunostomy time.

**Results:**

TSILC patients were older (29.86 ± 12.99 vs. 19.60 ± 13.62 months, *P* = 0.001), heavier (14.90 ± 5.07 vs. 9.22 ± 4.40 kg, *P* < 0.001), and had a different sex distribution (*P* = 0.030). After adjustment, total operative time did not differ significantly (adjusted *β*=8.75 min, 95% CI −12.10 to 29.60; *P* = 0.411), whereas jejunojejunostomy time remained longer with TSILC (adjusted β=12.02 min, 95% CI 3.02 to 21.02; *P* = 0.009). TSILC was associated with less blood loss, a shorter incision, and earlier first flatus and defecation (all adjusted *P* < 0.001); oral intake time did not differ significantly after adjustment (*P* = 0.568). Early complications occurred in 3/35 TSILC and 5/40 SILC patients. Thirty-three TSILC procedures were completed without an additional port or open conversion; one required an additional port and one required open conversion. Median follow-up was 40.0 months (IQR 21.5−59.5) for TSILC and 46.5 months (IQR 27.2−66.2) for SILC. The CUSUM curve peaked at case 17, suggesting relative stabilization after case 18.

**Conclusion:**

TSILC with intracorporeal hand-sewn Roux-en-Y reconstruction was technically feasible in selected children and showed a measurable learning curve. The findings suggest that TSILC may offer perioperative advantages in appropriately selected older and larger children.

## Introduction

Choledochal cyst is a common congenital biliary malformation in children and usually requires radical excision (1). Complete cyst excision with biliary reconstruction remains the accepted definitive treatment (2). With the development of pediatric minimally invasive surgery, laparoscopic choledochal cyst excision has become a widely used approach and has progressed from multiport laparoscopy to transumbilical single-incision-assisted procedures, total laparoscopic reconstruction, and robotic surgery (3–5). Compared with open surgery, laparoscopic surgery offers smaller wounds, faster recovery, and better cosmetic results. Single-incision techniques further reduce the number of visible abdominal scars and are particularly attractive in pediatric patients (6).

The conventional single-incision laparoscopic-assisted procedure usually requires enlargement of the umbilical incision. The jejunum is exteriorized, Roux-en-Y reconstruction is completed outside the abdomen, and the bowel is then returned before the laparoscopic field is re-established. This workflow is convenient and familiar, but it adds traction on the umbilical incision, exposes the bowel outside the abdomen, and requires repeated manipulation of the operative channel (7). Totally single-incision laparoscopic intracorporeal hand-sewn Roux-en-Y reconstruction avoids exteriorization of the bowel. It can reduce bowel traction and incision extension, and it allows the surgeon to check bowel orientation and the mesenteric defect under direct laparoscopic vision. However, this approach is technically demanding: jejunojejunostomy and hepaticojejunostomy must be completed through a single umbilical incision, with instrument crowding, limited angles, and a high demand for laparoscopic suturing (8). Although non-stapled total laparoscopic Roux-en-Y reconstruction has been reported in children with choledochal cysts (9), data on the learning curve of this procedure under totally single-incision conditions remain limited. We therefore reviewed 35 consecutive TSILC cases at our center and compared them with 40 contemporaneous conventional SILC cases, focusing on the learning curve, perioperative outcomes, and follow-up results.

## Materials and methods

### Patients

We retrospectively reviewed children who underwent laparoscopic choledochal cyst excision at Shanghai Children's Medical Center Guizhou Hospital from January 2018 to December 2024. Patients were grouped according to the reconstruction method: the TSILC group underwent totally single-incision laparoscopic intracorporeal hand-sewn Roux-en-Y reconstruction, whereas the SILC group underwent conventional single-incision laparoscopic-assisted Roux-en-Y reconstruction. There were 35 patients in the TSILC group and 40 in the SILC group. All 35 TSILC procedures were performed by the same senior pediatric laparoscopic surgeon, with a relatively stable assistant team, reducing the effect of surgeon variation on learning curve analysis. The patient selection and follow-up process is summarized in Figure 1.

**Figure 1:**
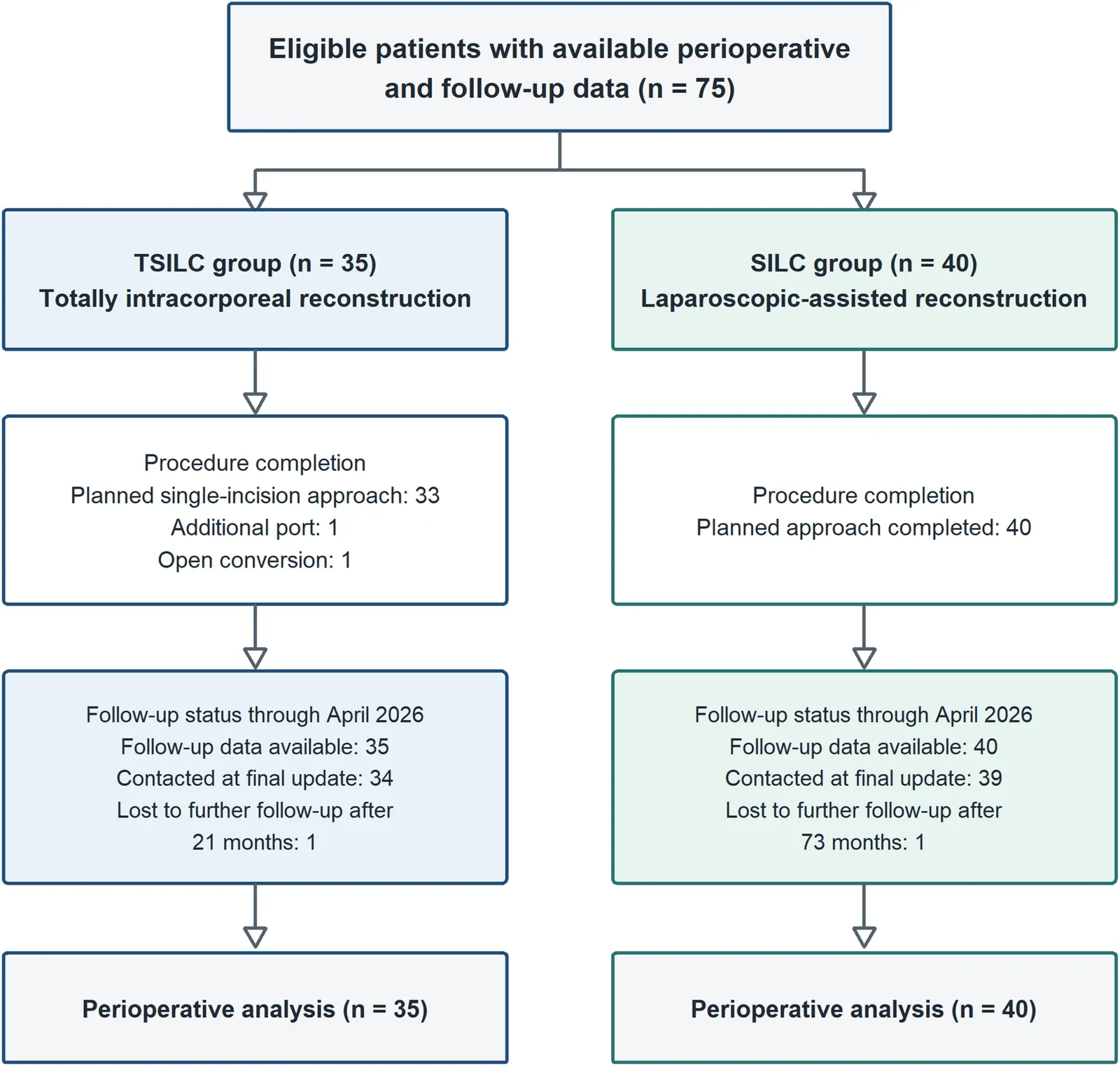
Patient selection and follow-up flow diagram. The diagram shows the eligible retrospective cohort, allocation by operative approach, procedure completion, follow-up status through April 2026, and the perioperative analysis populations. Follow-up was censored at the last successful contact for both patients lost to further follow-up; neither patient was considered under observation thereafter.

This was not a randomized study. During early TSILC adoption, our center preferentially selected older and heavier children because the larger working space was considered more favorable for intracorporeal suturing. Avoiding bowel exteriorization could also reduce the need for further extension of the umbilical incision. Consequently, TSILC patients were older than SILC patients (29.86 ± 12.99 vs. 19.60 ± 13.62 months, *P* = 0.001), heavier (14.90 ± 5.07 vs. 9.22 ± 4.40 kg, *P* < 0.001), and had a different sex distribution (*P* = 0.030). This selection strategy was considered in the adjusted analyses and interpretation of comparative outcomes.

Inclusion criteria were: (1) diagnosis of congenital choledochal cyst by ultrasonography, MRCP, or CT; (2) laparoscopic cyst excision with Roux-en-Y hepaticojejunostomy; and (3) available clinical and operative data with at least one postoperative follow-up assessment. Exclusion criteria were: (1) severe infection requiring emergency or staged surgery; (2) complex hepatobiliary malformations requiring special reconstruction; (3) previous complex upper abdominal surgery affecting the operative approach; and (4) missing key perioperative data or no postoperative follow-up information.

Patients were followed through outpatient visits or telephone interviews. Follow-up included clinical symptoms, liver function tests, ultrasonography, and MRCP when indicated. Follow-up was updated through April 2026. For patients who could not be contacted at the final update, follow-up duration was censored at the last successful contact. Their perioperative data and follow-up observations available up to that date were retained; they were not considered under observation thereafter.

The study was approved by the Medical Ethics Committee of Shanghai Children's Medical Center Guizhou Hospital, Shanghai Jiao Tong University School of Medicine (approval No. 2024−149; approval date: October 20, 2024). Because this was a retrospective study, informed consent was waived by the ethics committee.

### Surgical technique

In the TSILC group, patients were placed in a split-leg supine position. The primary surgeon stood between the legs, the camera assistant stood on the patient's left side, and the assistant stood on the right. A vertical umbilical incision was made through the umbilical ring into the abdominal cavity. A 5-mm trocar was inserted and fixed with a purse-string suture. Pneumoperitoneum was established at 8 to 12 mmHg (Figure 2). After subcutaneous dissection on both sides of the umbilical wound, two 3-mm trocars were inserted. The falciform ligament and gallbladder were suspended to expose the operative field. The gallbladder was dissected toward the junction of the cyst and cystic duct. The anterior wall of the cyst was opened for bile aspiration and decompression, and traction sutures were placed on the cyst to improve distal exposure. The cyst was dissected from distal to proximal. After management of the distal cyst at the pancreatic junction, posterior cyst dissection was continued upward to the level of the common hepatic duct, followed by cyst and gallbladder excision.

**Figure 2:**
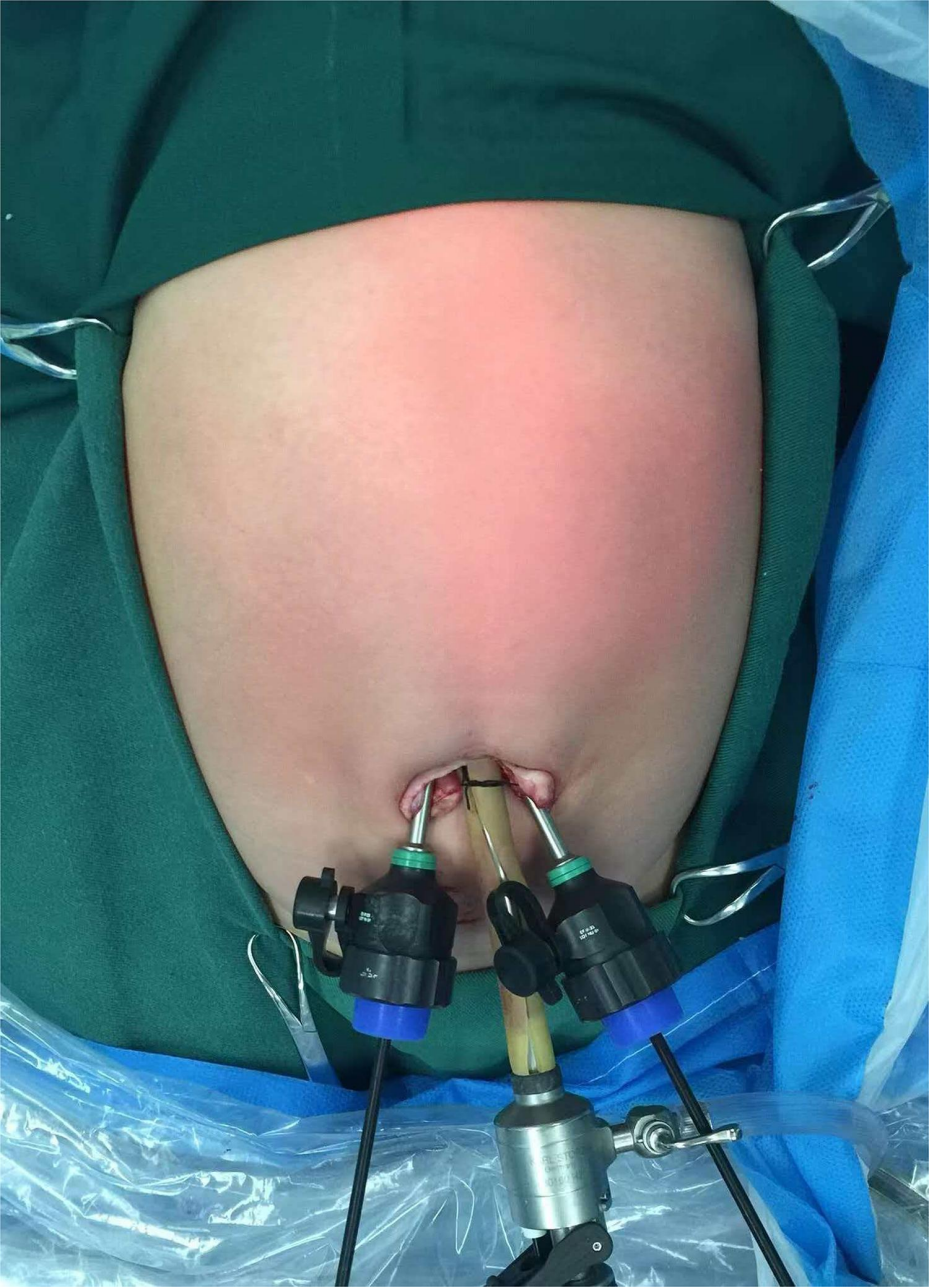
Umbilical trocar layout for TSILC. A 5-mm camera trocar was inserted through a vertical umbilical incision, and two 3-mm working trocars were placed on both sides of the same umbilical incision to create a transumbilical multi-channel working platform.

After cyst excision, the jejunum near the ligament of Treitz was lifted and oriented distally. The planned anastomotic segment was suspended to the abdominal wall to improve exposure. The mesentery and jejunum were divided, and the distal stump was closed with continuous sutures. The length of the biliary limb was individualized according to the distance from the hepatic hilum to the umbilicus, and the limb was suspended to the abdominal wall. The proximal jejunum and the biliary limb were fixed on the antimesenteric side. The side wall of the biliary limb was opened, and a hand-sewn end-to-side jejunojejunostomy was created with a double-armed 5−0 PDS suture using continuous single-layer suturing of the posterior and anterior walls. The mesenteric defect was then closed (Figure 3). The biliary limb was passed through the retrocolic route to the hepatic hilum without tension. According to the diameter of the hepatic duct opening, the antimesenteric side of the biliary limb was opened, and an end-to-side hepaticojejunostomy was performed with a double-armed 5-0 PDS suture in a continuous single-layer fashion. A drain was placed posterior to the anastomosis and brought out through the umbilical incision. The trocars were removed and the umbilicus was reconstructed (Figure 4). The complete operative process is shown in Supplementary Video 1.

**Figure 3:**
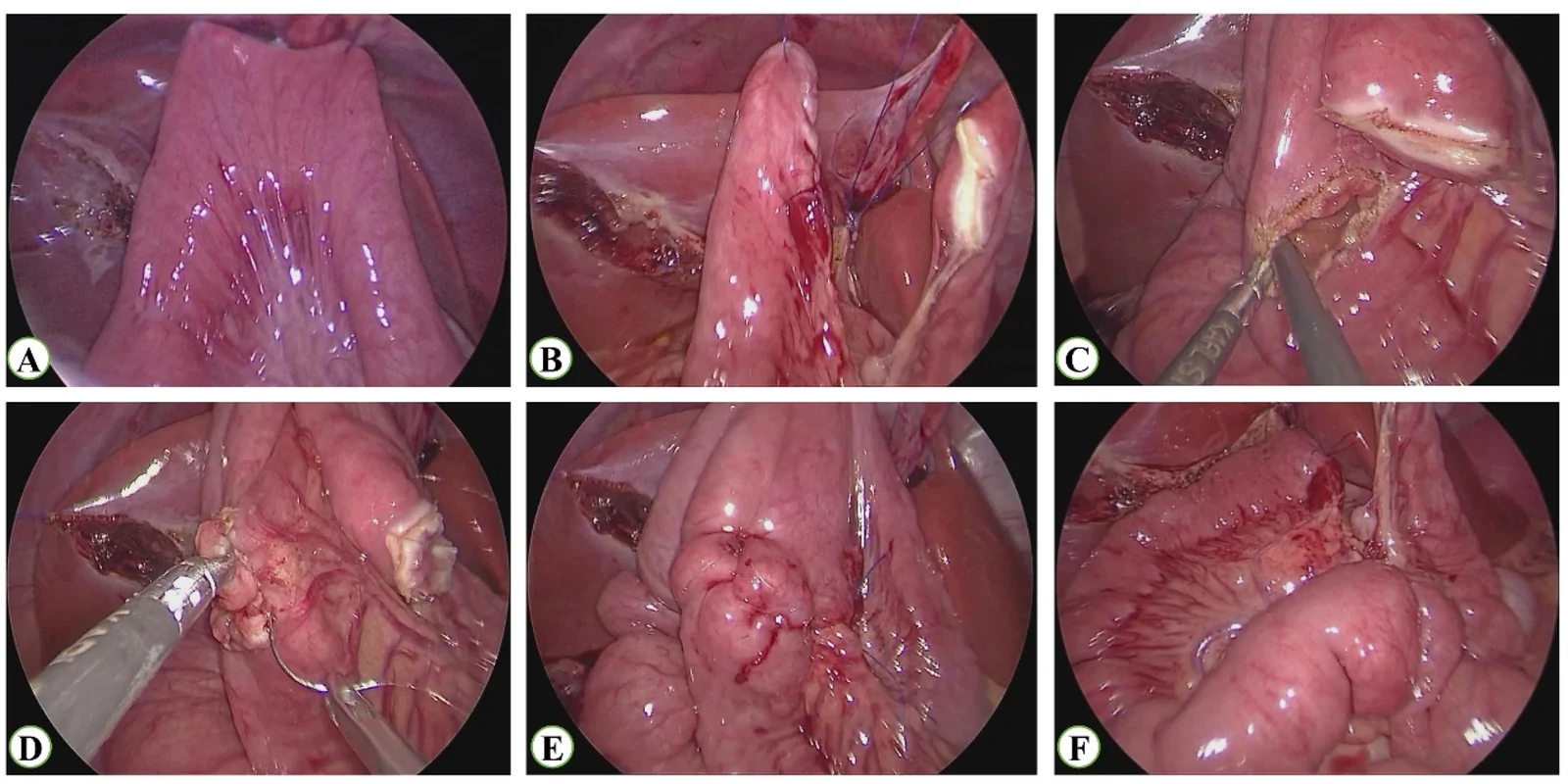
Key steps of intracorporeal hand-sewn jejunojejunostomy in TSILC. **(A)** The jejunal loop distal to the ligament of Treitz was straightened and suspended to the abdominal wall. **(B)** The jejunum and mesentery were divided, and the distal stump was closed with continuous sutures. **(C)** The Roux limb was measured and suspended, and the proximal jejunum was fixed to the Roux limb. **(D** and **E)** The posterior and anterior walls of the anastomosis were closed with continuous single-layer 5-0 PDS sutures. **(F)** Completion of the jejunojejunostomy and confirmation of the bowel orientation.

**Figure 4:**
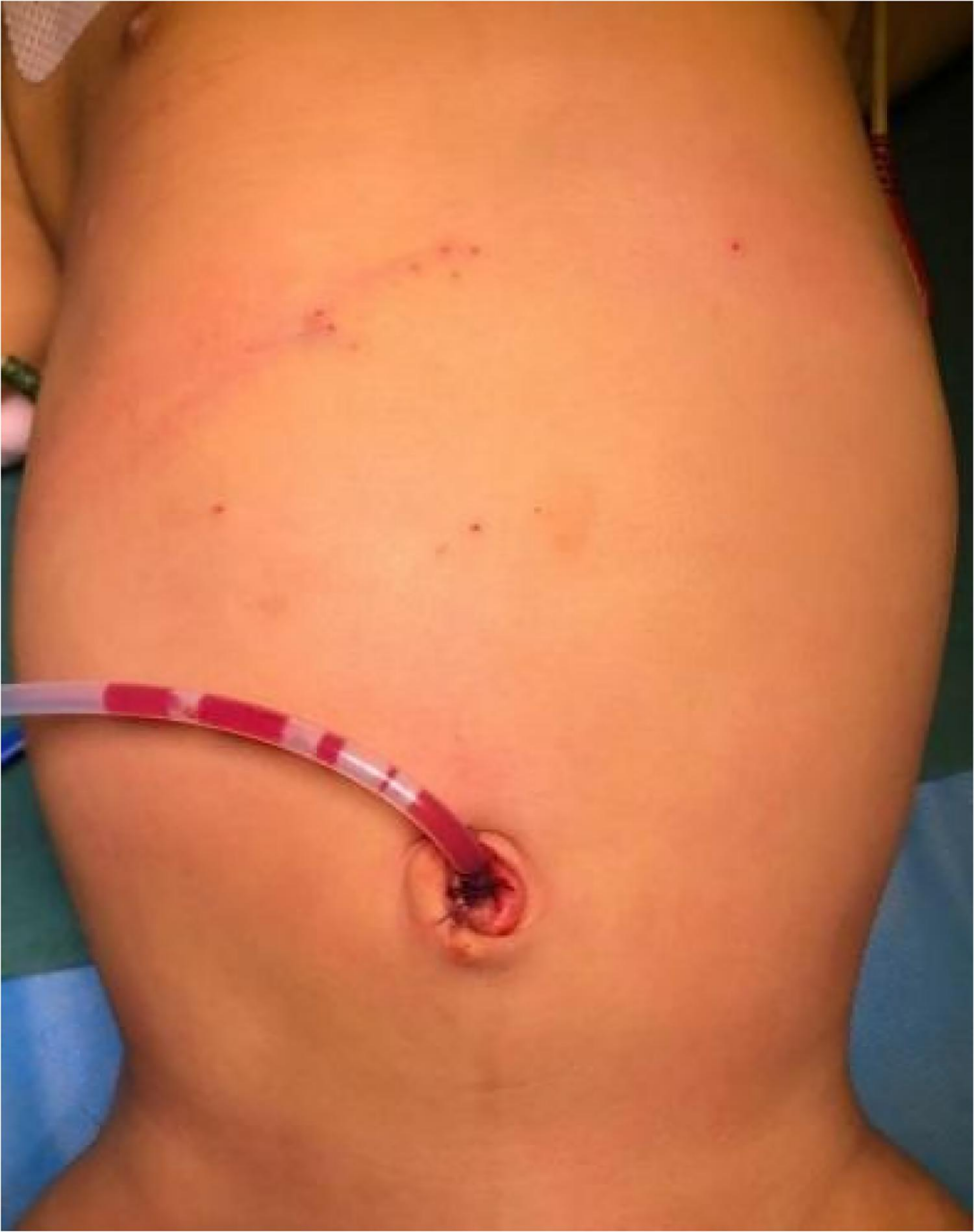
Umbilical incision management and drain exteriorization. After completion of hepaticojejunostomy, a drain was placed posterior to the anastomosis and exteriorized through the umbilical incision. The trocars were removed, and the umbilical incision was reconstructed.

In the SILC group, cyst excision was similar to that in the TSILC group. After cyst excision, the umbilical incision was enlarged, the jejunum was exteriorized, and Roux-en-Y reconstruction was performed extracorporeally with stapler assistance. The bowel was then returned to the abdominal cavity, the laparoscopic working channel was re-established, and hepaticojejunostomy and drainage were completed. In Table 2, jejunojejunostomy time in the SILC group was defined as the time from umbilical incision extension and exteriorization of the jejunum to completion of extracorporeal Roux-en-Y reconstruction, return of the bowel, and re-establishment of the laparoscopic working channel. This interval included incision extension, exteriorization, extracorporeal reconstruction, and return of the bowel, but did not include hepaticojejunostomy time. Postoperative management was the same in both groups. Oral feeding was started after the first passage of flatus. Drains were removed when daily output had decreased to 10 mL or less. Analgesics were administered as needed rather than routinely on a scheduled basis.

### Outcome measures and statistical analysis

Baseline variables, operative outcomes, postoperative recovery, complications, and follow-up results were collected. Baseline variables included sex, age, body weight, Todani type, cyst diameter, preoperative cholangitis, and preoperative pancreatitis. Operative variables included total operative time, anastomosis time, blood loss, incision length, additional port placement, and open conversion. Incision length was measured in centimeters and analyzed as an objective incision-related outcome rather than a validated cosmetic score. Intraoperative blood loss was obtained from the anesthesia record and was estimated from suction-canister volume after accounting for irrigation fluid, together with visual assessment of blood retained on operative gauze. Postoperative recovery variables included time to first flatus, time to first defecation, time to oral intake, drain removal time, and postoperative hospital stay. Early complications were defined as bile leakage, intra-abdominal infection, wound infection, intestinal obstruction, or reintervention during the postoperative period. Late complications included cholangitis, anastomotic stricture, intrahepatic bile duct dilatation, stone formation, or reoperation during follow-up.

Jejunojejunostomy time in the TSILC group was used for CUSUM analysis. The formula was CUSUM_*n* = *Σ*(X_i - *μ*), where X_i represents the jejunojejunostomy time of the i-th case and μ represents the mean time of all 35 TSILC cases. This step was selected because intracorporeal hand-sewn bowel reconstruction was the principal new technical component of TSILC, had a clearly defined start and end, and was less affected than total operative time by cyst inflammation, anatomic variation, and biliary dissection difficulty. Complication-based or composite learning-curve analyses were not performed because adverse events were infrequent and heterogeneous, making such curves statistically unstable in 35 cases. For descriptive phase analysis, the CUSUM peak at case 17 defined the main pre-peak/post-peak boundary. The pre-peak period was divided into phase I (cases 1-8) and phase II (cases 9-17), and the post-peak period into phase III (cases 18-26) and phase IV (cases 27-35), producing four approximately equal chronological blocks. These phases were descriptive and were not intended to define universal proficiency thresholds.

Because this was a retrospective study including all consecutive eligible patients during the study period, no *a priori* sample size calculation was performed. Descriptive and unadjusted analyses were performed using SPSS 22.0; multivariable models were fitted in Python 3.12 using statsmodels 0.14.6. Continuous variables were assessed for distributional form and are reported as mean ± standard deviation or median (interquartile range), as appropriate. Welch's *t* test was used for two-group comparisons because it does not assume equal variances and is more robust than the conventional Student t test when group variances or sample sizes differ. Categorical variables were compared using Fisher's exact test when event counts were small. Multivariable linear regression estimated adjusted mean differences for continuous operative and recovery outcomes, with SILC as the reference. Models included age, body weight, sex, Todani type, cyst diameter, preoperative cholangitis, and preoperative pancreatitis and used HC3 robust standard errors. Because age and weight were strongly correlated, sensitivity models containing age or weight separately were examined; the direction and significance pattern of the principal findings was unchanged. Sparse complication and conversion outcomes were not entered into multivariable models. Learning phases were compared using one-way ANOVA followed by pairwise Welch t tests with Holm adjustment. All tests were two-sided, and *P* < 0.05 was considered statistically significant.

## Results

### Baseline characteristics

A total of 75 children were included: 35 in the TSILC group and 40 in the SILC group. TSILC patients were older (29.86 ± 12.99 vs. 19.60 ± 13.62 months, *P* = 0.001), heavier (14.90 ± 5.07 vs. 9.22 ± 4.40 kg, *P* < 0.001), and had a different sex distribution (female/male: 23/12 vs. 35/5, *P* = 0.030). Todani type I/IV distribution was 27/8 versus 35/5 (*P* = 0.360), cyst diameter was 42.00 ± 12.75 versus 38.05 ± 11.84 mm (*P* = 0.171), preoperative cholangitis occurred in 7 versus 10 patients (*P* = 0.783), and preoperative pancreatitis in 5 versus 3 patients (*P* = 0.461) (Table 1).

**Table 1:** Preoperative clinical characteristics of the TSILC and SILC groups.

Characteristic	TSILC (*n* = 35)	SILC (*n* = 40)	*P* value
Female/male, n	23/12	35/5	0.030
Age (months)	29.86 ± 12.99	19.60 ± 13.62	0.001
Body weight (kg)	14.90 ± 5.07	9.22 ± 4.40	<0.001
Todani type I/IV, n	27/8	35/5	0.360
Cyst diameter (mm)	42.00 ± 12.75	38.05 ± 11.84	0.171
Preoperative cholangitis, *n* (%)	7 (20.0%)	10 (25.0%)	0.783
Preoperative pancreatitis, *n* (%)	5 (14.3%)	3 (7.5%)	0.461

Data are presented as mean ± SD or *n* (%), unless otherwise indicated. Continuous variables were compared using Welch's *t* test, and categorical variables were compared using Fisher's exact test. TSILC, totally single-incision laparoscopic intracorporeal hand-sewn Roux-en-Y reconstruction; SILC, conventional single-incision laparoscopic-assisted Roux-en-Y reconstruction.

### Operative outcomes

Unadjusted total operative time was 227.69 ± 28.17 min in the TSILC group and 221.32 ± 36.59 min in the SILC group (*P* = 0.399); the adjusted mean difference was 8.75 min (95% CI −12.10 to 29.60; *P* = 0.411). Jejunojejunostomy time was longer with TSILC (51.86 ± 16.84 vs. 36.95 ± 8.13 min, *P* < 0.001), and the difference remained after adjustment (adjusted *β*=12.02 min, 95% CI 3.02 to 21.02; *P* = 0.009). Hepaticojejunostomy time did not differ significantly (44.77 ± 6.07 vs. 45.88 ± 9.57 min; adjusted *P* = 0.299). TSILC was associated with less blood loss (7.14 ± 2.72 vs. 10.88 ± 3.22 mL; adjusted *β*=-4.77 mL, 95% CI −6.96 to −2.59; *P* < 0.001) and a shorter incision (1.68 ± 0.22 vs. 3.59 ± 0.56 cm; adjusted *β*=-1.96 cm, 95% CI −2.33 to −1.59; *P* < 0.001). Thirty-three of 35 TSILC procedures were completed without an additional port or open conversion. One patient required an additional port and a separate patient required open conversion; all 40 SILC procedures were completed without either event (Table 2).

**Table 2:** Operative outcomes and multivariable-adjusted comparisons.

Outcome	TSILC (*n* = 35)	SILC (*n* = 40)	Unadjusted *P*	Adjusted β (95% CI)	Adjusted *P*
Total operative time (min)	227.69 ± 28.17	221.32 ± 36.59	0.399	+8.75 (−12.10 to 29.60)	0.411
Jejunojejunostomy time (min)	51.86 ± 16.84	36.95 ± 8.13	<0.001	+12.02 (3.02 to 21.02)	0.009
Hepaticojejunostomy time (min)	44.77 ± 6.07	45.88 ± 9.57	0.548	−3.12 (−9.00 to 2.77)	0.299
Blood loss (mL)	7.14 ± 2.72	10.88 ± 3.22	<0.001	−4.77 (−6.96 to −2.59)	<0.001
Incision length (cm)	1.68 ± 0.22	3.59 ± 0.56	<0.001	−1.96 (−2.33 to −1.59)	<0.001
Completion without additional port or open conversion, *n* (%)	33 (94.3%)	40 (100.0%)	0.214	NA	NA
Additional port, *n* (%)	1 (2.9%)	0 (0.0%)	0.467	NA	NA
Open conversion, *n* (%)	1 (2.9%)	0 (0.0%)	0.467	NA	NA

Continuous data are mean ± SD. Unadjusted *P* values were obtained using Welch's *t* test. Adjusted β values represent the mean difference for TSILC versus SILC (reference) from multivariable linear regression with HC3 robust standard errors, adjusted for age, body weight, sex, Todani type, cyst diameter, preoperative cholangitis, and preoperative pancreatitis. Positive values indicate higher values in the TSILC group. Sparse categorical outcomes were compared using Fisher's exact test and were not entered into multivariable models. CI, confidence interval; NA, not applicable.

### Postoperative recovery and complications

Time to first flatus (20.28 ± 2.70 vs. 28.92 ± 4.55 h) and first defecation (28.61 ± 4.05 vs. 37.33 ± 4.80 h) were shorter in the TSILC group. These associations remained after adjustment (adjusted *β*=-7.11 h, 95% CI −9.71 to −4.50, and −7.30 h, 95% CI −10.31 to −4.28, respectively; both *P* < 0.001). Oral intake occurred earlier in the unadjusted comparison (1.95 ± 0.52 vs. 2.19 ± 0.53 d, *P* = 0.048), but this difference was not significant after adjustment (adjusted *β*=0.12 d, 95% CI −0.28 to 0.52; *P* = 0.568). Drain removal time and postoperative hospital stay did not differ significantly. Early complications occurred in 3 TSILC patients and 5 SILC patients (*P* = 0.716); late complications occurred in 2 and 3 patients, respectively (*P* = 1.000). Median follow-up was 40.0 months (IQR 21.5-59.5; range 6-79) for TSILC and 46.5 months (IQR 27.2-66.2; range 9-84) for SILC (*P* = 0.254). One patient in each group was lost to further follow-up; follow-up was censored at the last successful contact at 21 months in the TSILC group and 73 months in the SILC group. Perioperative data and follow-up observations through those dates were retained, whereas outcomes thereafter were unknown. Most children had normal liver function and patent anastomoses on imaging (Table 3 and Figure 5).

**Table 3:** Postoperative recovery, complications, and multivariable-adjusted comparisons.

Outcome	TSILC (*n* = 35)	SILC (*n* = 40)	Unadjusted P	Adjusted β (95% CI)	Adjusted P
Time to first flatus (h)	20.28 ± 2.70	28.92 ± 4.55	<0.001	−7.11 (−9.71 to −4.50)	<0.001
Time to first defecation (h)	28.61 ± 4.05	37.33 ± 4.80	<0.001	−7.30 (−10.31 to −4.28)	<0.001
Time to oral intake (d)	1.95 ± 0.52	2.19 ± 0.53	0.048	+0.12 (−0.28 to 0.52)	0.568
Drain removal time (d)	3.26 ± 0.91	3.06 ± 1.45	0.463	+0.11 (−0.80 to 1.02)	0.816
Postoperative hospital stay (d)	5.93 ± 1.52	6.37 ± 1.84	0.257	−1.14 (−2.32 to 0.03)	0.056
Early complications, *n* (%)	3 (8.6%)	5 (12.5%)	0.716	NA	NA
Late complications, *n* (%)	2 (5.7%)	3 (7.5%)	1.000	NA	NA
Reoperation, *n* (%)	1 (2.9%)	2 (5.0%)	1.000	NA	NA
Follow-up duration, months	40.0 (21.5–59.5; range 6–79)	46.5 (27.2–66.2; range 9–84)	0.254	NA	NA
Lost to further follow-up, *n* (%)	1 (2.9%)	1 (2.5%)	1.000	NA	NA

Continuous recovery outcomes are mean ± SD. Unadjusted *P* values were obtained using Welch's *t* test. Adjusted β values represent the mean difference for TSILC versus SILC (reference) from multivariable linear regression with HC3 robust standard errors, adjusted for age, body weight, sex, Todani type, cyst diameter, preoperative cholangitis, and preoperative pancreatitis. Complications, reoperation, and loss to further follow-up were compared using Fisher's exact test and were not adjusted because of sparse events. Follow-up duration is median (interquartile range; range) and was compared using the Mann–Whitney U test. For patients lost to further follow-up, follow-up duration was censored at the last successful contact. CI, confidence interval; NA, not applicable.

**Figure 5:**
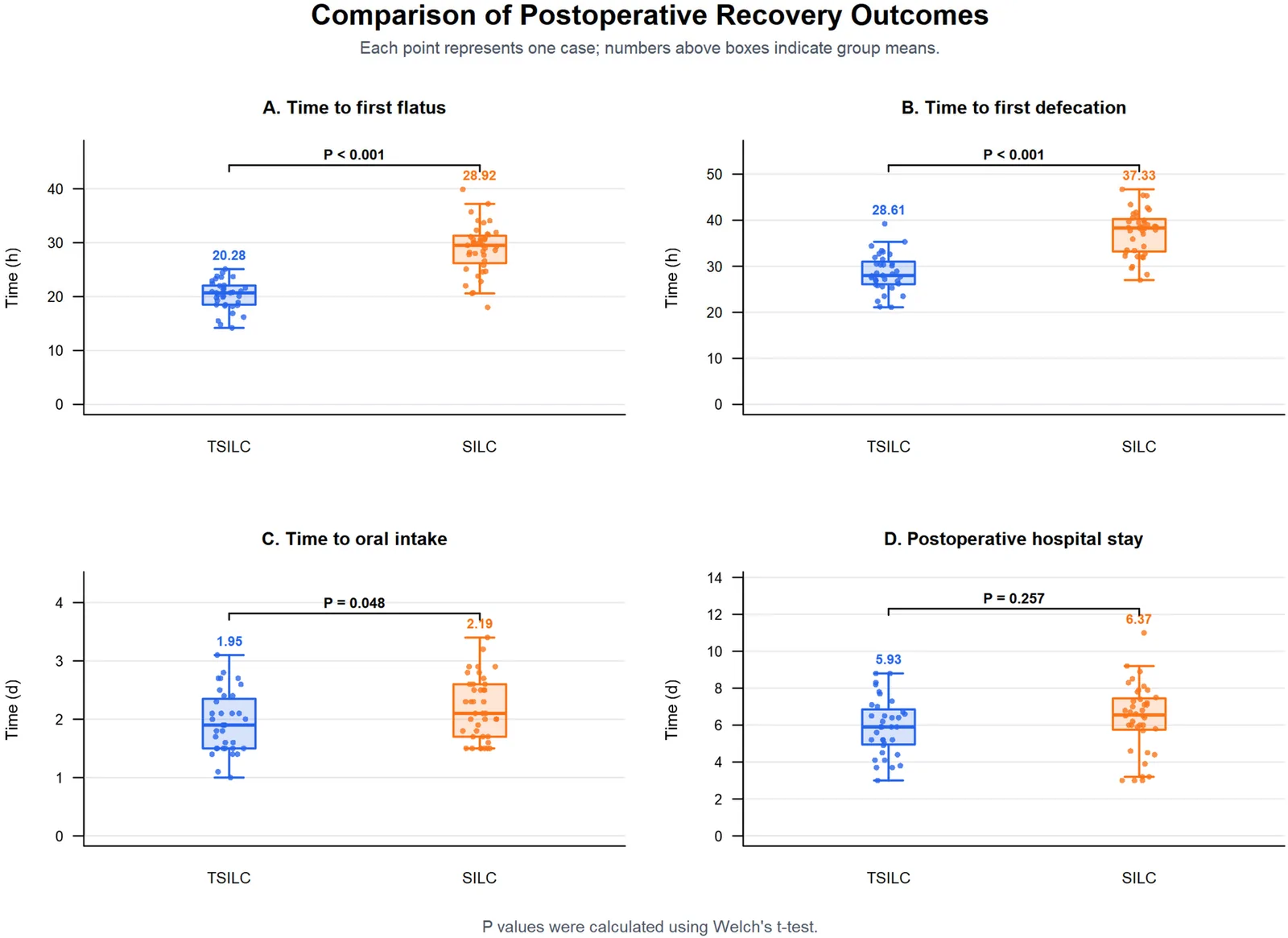
Comparison of postoperative recovery outcomes between the TSILC and SILC groups. **(A)** Time to first flatus. **(B)** Time to first defecation. **(C)** Time to oral intake. **(D)** Postoperative hospital stay. Each point represents one patient, and numbers above boxes indicate group means. Displayed *P* values are unadjusted Welch t-test results; multivariable-adjusted estimates are reported in Table 3.

### Learning curve

Mean jejunojejunostomy time among the 35 TSILC cases was 51.86 min. The CUSUM curve peaked at case 17 (CUSUM=239.43) and then declined, suggesting relative stabilization after case 18 (Figure 6). Total operative time was 254.38 ± 20.35, 243.89 ± 16.00, 217.56 ± 10.33, and 197.89 ± 22.92 min in phases I-IV, respectively. Jejunojejunostomy time was 71.88 ± 12.73, 60.67 ± 8.35, 43.00 ± 5.36, and 34.11 ± 5.84 min, and hepaticojejunostomy time was 51.88 ± 2.80, 48.56 ± 1.67, 42.33 ± 2.12, and 37.11 ± 1.76 min. Overall differences across phases were significant for all three outcomes (all one-way ANOVA *P* < 0.001); Holm-adjusted *post-hoc* comparisons are reported in Table 4.

**Figure 6:**
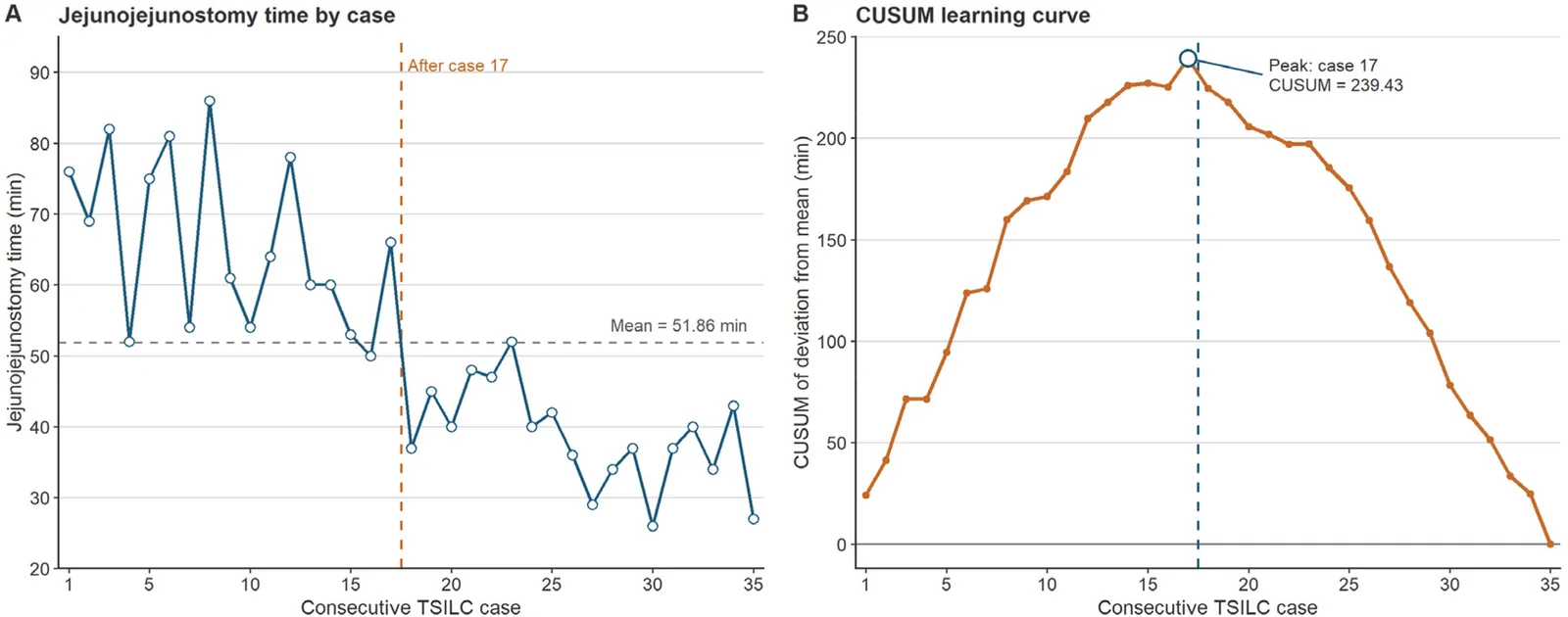
Learning curve analysis of TSILC intracorporeal jejunojejunostomy. **(A)** Case-by-case jejunojejunostomy time. The horizontal dashed line indicates the overall mean (51.86 min), and the vertical dashed line separates cases 1-17 from cases 18-35. **(B)** CUSUM curve based on jejunojejunostomy time. The curve peaked at case 17 (CUSUM=239.43) and declined thereafter, suggesting relative stabilization after case 18.

**Table 4:** Operative time and *post-hoc* comparisons across learning phases in the TSILC group.

Panel A. Phase summary and overall ANOVA					
Outcome	Phase I (*n* = 8)	Phase II (*n* = 9)	Phase III (*n* = 9)	Phase IV (*n* = 9)	Overall P
Total operative time (min)	254.38 ± 20.35	243.89 ± 16.00	217.56 ± 10.33	197.89 ± 22.92	<0.001
Jejunojejunostomy time (min)	71.88 ± 12.73	60.67 ± 8.35	43.00 ± 5.36	34.11 ± 5.84	<0.001
Hepaticojejunostomy time (min)	51.88 ± 2.80	48.56 ± 1.67	42.33 ± 2.12	37.11 ± 1.76	<0.001

Panel B. Holm-adjusted *P* values for pairwise comparisons			
Comparison	Total operative time	Jejunojejunostomy time	Hepaticojejunostomy time
I vs. II	0.262	0.056	0.014
I vs. III	0.004	<0.001	<0.001
I vs. IV	<0.001	<0.001	<0.001
II vs. III	0.004	<0.001	<0.001
II vs. IV	0.001	<0.001	<0.001
III vs. IV	0.077	0.008	<0.001

Data are mean ± SD. Phase I comprised cases 1–8, Phase II cases 9–17, Phase III cases 18–26, and Phase IV cases 27–35. Overall *P* values in Panel A were obtained using one-way ANOVA. Panel B reports all Holm-adjusted *P* values from pairwise Welch t tests. The CUSUM curve based on jejunojejunostomy time peaked at case 17 (CUSUM=239.43), indicating relative stabilization after case 18.

## Discussion

Pediatric choledochal cyst surgery has progressed from multiport laparoscopy to single-incision-assisted, total laparoscopic, and robotic reconstruction (10, 11). The conventional single-incision-assisted approach reduces the number of ports but often requires enlargement of the umbilical incision for extracorporeal Roux-en-Y reconstruction (12–14). Intracorporeal reconstruction avoids bowel exteriorization but demands advanced suturing through a restricted single-incision working axis (15, 16).

In the adjusted analysis, total operative time and hepaticojejunostomy time were similar between groups, whereas jejunojejunostomy time remained longer with TSILC. TSILC was also associated with less blood loss, a shorter incision, and earlier flatus and defecation (16, 17). Oral intake time did not differ significantly after adjustment. These findings suggest that TSILC may offer several perioperative advantages in appropriately selected older and larger children.

The main technical challenge was intracorporeal hand-sewn jejunojejunostomy. Unlike the assisted method, TSILC requires bowel division, limb measurement, orientation, anastomosis, and mesenteric defect closure with coaxial instrument interference and limited triangulation. The adjusted increase of approximately 12 min in jejunojejunostomy time is therefore clinically understandable. As experience accumulated, total operative time declined and the other steps became more efficient, which may explain why overall operative time remained similar despite the longer jejunojejunostomy.

A practical advantage of TSILC is that reconstruction remains under direct laparoscopic vision. The surgeon can confirm bowel orientation, Roux-limb routing, and mesenteric defect closure without exteriorizing or repeatedly manipulating the bowel (20). Incision length was also clearly reduced after adjustment (adjusted mean difference, −1.96 cm; 95% CI, −2.33 to −1.59; *P* < 0.001), and representative follow-up photographs showed well-healed scars concealed within the umbilical fold (Figure 7). These findings support the practical benefit of preserving a small umbilical access incision.

**Figure 7:**
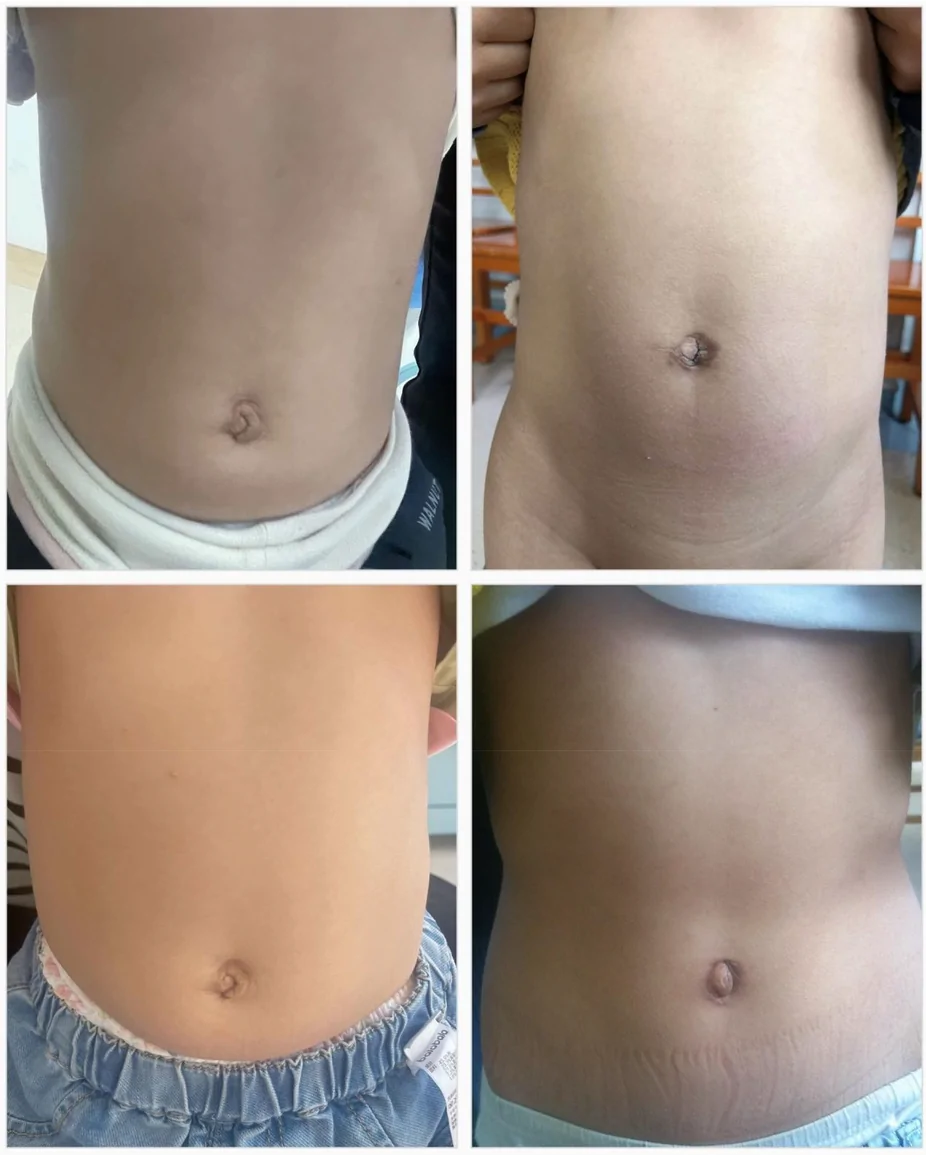
Postoperative single-incision appearance. Representative follow-up photographs showing well-healed umbilical incisions after TSILC. These images are descriptive. Incision length was measured separately as an objective incision-related outcome, but no validated cosmetic score or parent-/patient-reported cosmetic outcome was collected.

CUSUM has previously been used to evaluate proficiency in laparoscopic choledochal cyst excision and Roux-en-Y reconstruction (21). Jejunojejunostomy time was selected for CUSUM analysis because it represents the principal new technical step of TSILC, has a clear start and end, and can be recorded consistently. The curve peaked at case 17 and declined thereafter. Together with the progressive reduction in total operative, jejunojejunostomy, and hepaticojejunostomy times across the four phases (Table 4), this pattern indicates relative technical stabilization after case 18. The four chronological blocks were used to show the gradual change around the CUSUM peak.

During early adoption, TSILC was used mainly in older and heavier children. Their larger abdominal working space and bowel caliber made intracorporeal suturing easier (16). At the same time, exteriorizing the thicker bowel and mesentery through the umbilicus could require greater incision extension and traction. In smaller children, the bowel could usually be brought out through only a slight extension of the umbilical incision, whereas intracorporeal suturing was more difficult and offered less practical benefit. We therefore viewed TSILC as a selective option rather than a routine replacement for SILC.

The observed early and late complication rates were similar between groups. Previous large laparoscopic series and reports of total intracorporeal reconstruction have also described low conversion rates and acceptable short- to intermediate-term outcomes (18, 19). In the TSILC group, one patient required an additional port and another required open conversion. These events emphasize that secure exposure and reconstruction take priority over maintaining a single incision. Additional access or conversion should be used promptly when suturing, perfusion, bowel orientation, or anastomotic tension is uncertain.

This study has several limitations. It was a single-center retrospective study with a small sample, and deliberate patient selection resulted in substantial baseline imbalance. Multivariable adjustment and sensitivity analyses cannot fully exclude residual confounding, particularly for recovery outcomes. The small number of adverse events precluded firm conclusions about safety equivalence and made complication-based or composite learning-curve analysis unreliable. Although incision length was measured objectively, no validated scar scale or parent-/patient-reported cosmetic outcome was collected. One patient in each group was lost to further follow-up; although observations through the last successful contact were available, subsequent late events could not be ascertained. Longer follow-up is needed for late biliary complications (10, 11), and the learning curve of one experienced surgeon may not be generalizable to other teams or lower-volume centers.

In conclusion, totally single-incision laparoscopic choledochal cyst excision with intracorporeal hand-sewn Roux-en-Y reconstruction was technically feasible in selected children and showed relative stabilization after approximately 18 cases. Compared with conventional single-incision-assisted reconstruction, TSILC was associated with a smaller incision, less blood loss, and earlier flatus and defecation after adjustment, although intracorporeal jejunojejunostomy remained technically demanding. TSILC may be a useful option for appropriately selected older and larger children; larger prospective studies are needed to define its indications and confirm these findings.

## Data Availability

The raw data supporting the conclusions of this article will be made available by the authors, without undue reservation.
